# Demographics, Socioeconomic Context, and the Spread of Infectious Disease: The Case of COVID-19

**DOI:** 10.3390/ijerph19042206

**Published:** 2022-02-15

**Authors:** Yung-Hsiang Ying, Wen-Li Lee, Ying-Chen Chi, Mei-Jung Chen, Koyin Chang

**Affiliations:** 1 Department of Business Administration, National Taiwan Normal University, Taipei 106, Taiwan; yying@gapps.ntnu.edu.tw; 2 Department of Healthcare Information and Management, Ming Chuan University, Taoyuan 333, Taiwan; wllee@mail.mcu.edu.tw (W.-L.L.); janechi@mail.mcu.edu.tw (Y.-C.C.); 3 Department of Biomedical Engineering, Ming Chuan University, Taoyuan 333, Taiwan; mjchen@mail.mcu.edu.tw; 4 Harris School of Public Policy, University of Chicago, Chicago, IL 60611, USA

**Keywords:** infectious diseases, socioeconomics, quantile regression, mixed effect model

## Abstract

Importance: Due to the evolving variants of coronavirus disease 2019 (COVID-19), it is important to understand the relationship between the disease condition and socioeconomic, demographic, and health indicators across regions. Background: Studies examining the relationships between infectious disease and socioeconomic variables are not yet well established. Design: A total of 3042 counties in the United States are included as the observation unit in the study. Two outcome variables employed in the study are the control of disease spread and infection prevalence rates in each county. Method: Data are submitted to quantile regression, hierarchical regression, and random forest analyses to understand the extent to which health outcomes are affected by demographics, socioeconomics, and health indicators. Results: Counties with better control of the disease spread tend to have lower infection rates, and vice versa. When measuring different outcome variables, the common risk factors for COVID-19 with a 5% level of statistical significance include employment ratio, female labor ratio, young population ratio, and residents’ average health risk factors, while protective factors include land size, housing value, travel time to work, female population ratio, and ratio of residents who identify themselves as mixed race. Conclusions: The implications of the findings are that the ability to maintain social distancing and personal hygiene habits are crucial in deterring disease transmission and lowering incidence rates, especially in the early stage of disease formation. Relevant authorities should identify preventive factors and take early actions to fight infectious diseases in the future.

## 1. Background

Infectious diseases have spread quickly throughout the world in recent decades. The rapidity with which we travel across borders and continents has fueled disease outbreaks, such as avian influenza, Middle East respiratory syndrome-coronavirus (MERS-CoV), and the most current and ongoing outbreak of coronavirus disease 2019, known as COVID-19. While a renewed focus on pandemic planning has been established, understanding how the disease spreads and how it is affected by health indicators and socioeconomic factors have yet to be investigated and are of great interest to many healthcare professionals and social scientists.

The hypothesis that socioeconomic determinants of health, such as poverty, race, ethnicity, social marginalization, and environment, are linked to infectious diseases, including influenza, malaria, tuberculosis, Ebola, and other diseases, has been widely acknowledged [1,2,3]. The WHO Commission on the Social Determinants of Health, Closing the Gap, also explicitly stated that health inequalities may fuel many infectious diseases [4]. However, the link between acute respiratory tract infections (which is most relevant in pandemic preparedness planning) and poverty has only been reviewed and acknowledged very recently [5].

Cross-country comparisons of health outcomes have always focused more on nonrespiratory types of infectious diseases. Within countries, studies of health disparities are primarily concentrated on life expectancy, cancers, obesity, and chronic diseases such as cardiovascular or diabetes, that are attributed to a poor diet or inadequate lifestyle [6,7]. Among the few studies that examine respiratory diseases, Blumenshine et al. (2008) [8] discussed the potential causes of disparities in the U.S. during an influenza outbreak using a disparities model [9]. By establishing the linkages between differential exposure to the influenza virus and differential access to health care once the disease has developed, their results indicated the need for careful and systematic planning to minimize social disparities when faced with a pandemic.

**Figure 1:**
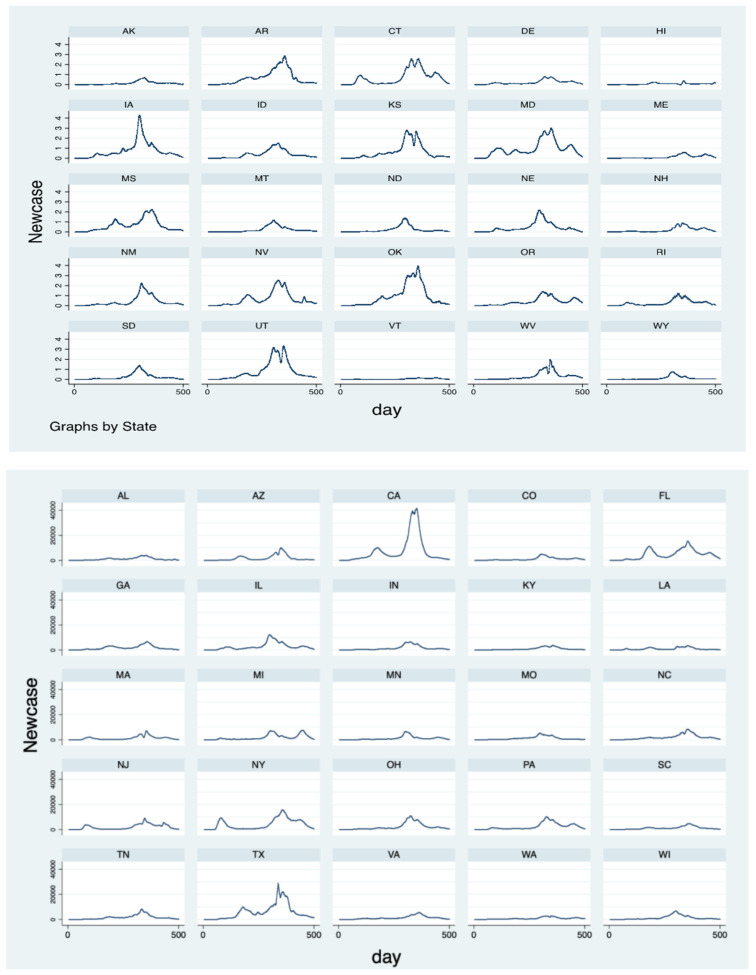
Number of new cases in each state. Note: The horizontal axis indicates the number of days since 22 January 2020. The vertical axis is measured in thousands.

**Figure 2:**
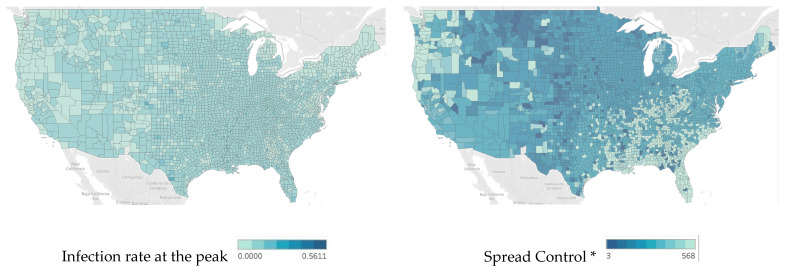
Speed of COVID-19 Spread in the Pre-Delta Strain Period and Infection Outcomes by County. Note: * Spread control is measured by the number of days required for the infection rate to reach 3%. Fewer days represent faster speed or poorer control of spread.

As a continuation of studies of health disparities in the context of infectious disease, this study aims to investigate the extent to which socioeconomics and health disparities contribute to the variation in the conditions of those who contract the infectious disease. The COVID-19 pandemic is still raging. With evolving variants emerging, this study focuses on the pre-Delta strain period, from 22 January 2020, to 15 March 2021, and discusses disease progression: first, the rapidity of COVID-19 spread across each U.S. county, and second, the counties’ infection (or prevalence) rates. In the pre-Delta period, the new confirmed case numbers in each county peaked and then decreased after vaccines became available. The county figures aggregated at the state level are shown in Figure 1. This phenomenon provides a scenario for understanding infectious diseases starting from the outbreak until the vaccine gradually became readily available. Geographic variation in the disease impact is conspicuous, as shown in Figure 2, which shows the speed of the disease spread and severity level of the infection (deeper colors indicate faster spread and a more severe infection status). These two indicators are obtained in the study to reveal the relationships between the demographics, socioeconomic variables, health indicators, and other relevant control variables within each county. Using these data collected from different sources, we aim to investigate the extent to which infectious disease conditions are affected by geographical variations in socioeconomics, demographics, healthcare indicators, and characteristics of the healthcare institutions in the counties. The results of this study will provide implications for future policy amendments and recommendations for measures that relevant authorities should implement to prepare for similar pandemics.

### 1.1. Literature Review

#### 1.1.1. Inequality in U.S. Healthcare

Differential healthcare seeking behaviors may exist for individuals with different income levels, potentially driven by differential access to healthcare. These differences might lead to delays in seeking care in response to respiratory infection [10], as well as differences in the quality of care available. Less access to healthcare may result in uncontrolled chronic conditions, such as pneumonia, asthma, and septicemia, and hence more severe diseases [11]. These conditions may lead to differences in the rates of antiviral prescriptions [12] and differential outcomes that require hospitalization, both of which contribute to the social consequences of ill health and further social stratification [9]. Income-related disparities in access to care are far wider in the United States than in other wealthy countries. Thirty-nine percent of Americans with a below-average income reported not seeing a doctor for a medical problem because of cost, compared with 7% of low-income Canadians and 1% of those in the UK [13]. Disparities in access are largely due to high rates of no insurance or insufficient insurance among low-income Americans. This group of people is more likely than adequately insured people to forgo needed medical services and medications because of cost. This condition is especially severe for millions of uninsured Americans with chronic conditions [14]. For infectious diseases, strong correlations between poverty and tubercular disease, influenza, acute respiratory tract infection, and acute respiratory infection are well documented [5].

However, researchers do not find a strong hazardous effect of income inequality on all-cause mortality using the U.S. data when controlling for income, education, race, and urbanization [15,16], which is only observed for homicides and, to a lesser extent, infant mortality and deaths from accidents. In general, although income is positively related to health, income inequality does not contribute to a higher population mortality rate. Thus, in this study, we include income level as a risk factor, but not income inequality.

#### 1.1.2. Health Inequality and Race

Health disparities take on many forms for racial and ethnic minorities, including infant mortality, chronic disease, and premature death, compared to the rates among ethnic groups [17,18]. Other conditions, such as obesity and related chronic diseases and debilitating conditions, also disproportionately affect racial and ethnic minorities, which have major implications for the quality of life and wellbeing of these population groups. For example, Asians had the lowest prevalence rate (8.6%) of obesity in the U.S., and Hispanic children had the highest prevalence (21.9%) from 2011 to 2014 (NCHS, 2016) [19]. African Americans were 30% and 100% more likely to die prematurely from heart disease and stroke, respectively, in 2010 than their white counterparts (HHS, 2016) [20]. African Americans have the highest mortality rate for all cancers combined compared with any other racial and ethnic group [21]. As race plays a role in health inequality, it must be included as a factor when determining the spread and severity of COVID-19 across counties.

#### 1.1.3. Health Inequality and Education

Education and health are both considered indicators of the quality of human capital that can be invested and are linked to income level. The existing health economics literature suggests that the causal effect running from income to health is indirect and might be mediated by the purchase of healthcare services, suggesting that the correlation between income and health is potentially driven by factors such as education or rates of time preference [22,23,24]. Those who have a stronger desire for current consumption are likely to fail to make investments to protect their health and fail to obtain the education and skills needed to generate higher earnings [25]. Even with the endogeneity between these variables, income and education are still considered independent protective factors for self-reported health status [26]. Researchers apply state or metropolitan data and find relationships among mortality, income, and education. Specifically, average education drives average income and modulates the effect on mortality and even shifts it to a risk factor [27,28,29]. However, the conflict between the individual and aggregate data remains unresolved. Education is included in the study to control this underlying effect.

The aforementioned discussion implies that disadvantaged populations might be particularly vulnerable and susceptible to pandemics and crowd hospital wards, placing medical personnel at great risk. Understanding the spread of highly contagious diseases and considering socioeconomic factors are very important in policy implications. Recommendations for policies to prepare for and respond to a respiratory disease pandemic are a crucial need [30].

## 2. Research Method

According to a previous study that explains the variations in health status mediated by socioeconomic factors [31], this research project investigates the population outcome, denoted as Y for district (county) I across the United States. The variation related to socioeconomics (S), demographics and geographics (D), and health-related indicators (H) is calculated using Equation (1) as follows.

(1) $$Y_{i}=\beta_{0}+\beta_{1}S_{i}+\beta_{2}D_{i}+\beta_{3}H_{i}+\varepsilon_{i}$$

The population outcome Y in our study is the COVID-19 conditions, which are measured in two ways: control of disease spread and the severity of the infection. Explanations are provided below.

### 2.1. Spread of Infectious Disease

Traditionally, the basic reproduction number (R) is adopted in the field of public health to show the speed of infection for a disease. It is the average number of people who will be infected by a single infectious person over the course of his or her illness. This number, however, is constantly changing and is highly sensitive to short-term conditions and the specific methods of computation. Alternatively, in this study, we measured the number of days for the disease to spread from the first incident to the first day the data are available, depending on which came first, to the day that the infection rate reached 3% of the county population. This measure provides a direct indicator of how rapidly the virus is spreading. A longer time to reach 3% implies better control of disease spread in the county. The reason we chose 3% as the benchmark was because only 12 counties of the 3138 counties included in our study had a maximum infection rate of less than 3% in the pre-Delta period of the pandemic [1]. Even counties with a maximum infection rate of less than 3% have a value close to 3%. Thus, it could be an objective indicator of spread control.

### 2.2. Infection Severity

The direct measure of the severity of a disease in an area is the incidence or prevalence rate of the disease. This study employs the prevalence rate by accumulating positive confirmed case numbers each day divided by population numbers in the U.S. counties. The number of new cases increases and decreases, but the cumulative case numbers plateau when the disease condition is alleviated. For each county, the prevalence rate is computed on the day of the peak of newly confirmed cases as the indicator of the severity in each county. In the few counties where the newly confirmed cases did not reach the summit in the pre-Delta period, prevalence rates were computed at the end of the study period, 15 March 2021.

### 2.3. Study Period

Our study period, described as the COVID-19 pre-Delta period, is from 22 January 2020 to 15 March 2021. The start date is based on the availability of the daily statistics of COVID-19 released by USAFacts. The end date is the trough of the trend of newly confirmed cases in most of the states, representing the end of the first wave of COVID-19 and the beginning of the spread of the Delta variant.

### 2.4. Explanatory Variables

#### 2.4.1. Socioeconomics

Health inequality is best known to be attributed to income disparity, as stated in the previous section. Other associated factors, such as employment conditions and urbanization of the district, are potential determinants to be included in the control covariates. Housing value, broadband internet coverage, and the female labor force participation rate are included as controls related to the urbanization of the counties.

#### 2.4.2. Herfindahl–Hirschman Index (HHI)

The HHI is a measure of industrial competitiveness. Suppliers’ behaviors are substantially influenced by the market condition, and an interdependent relationship exists among institutions. In economic theory, quality is one of the components of nonprice competition, which might be a focal point of healthcare institutions when publicizing their brand names in the industry. Institutions with higher market power can manipulate their prices and quality to differentiate themselves in the broad band of services in the market. In contrast, firms facing fierce competition may be more cost conscious and maintain a minimum level of required quality. As a result, the HHI may be an important determinant of the variation in the quality of healthcare institutions across regions. This index is not readily available from government publications; thus, we imputed the figure based on the number of patients served by each of the healthcare institutions listed on the Center for Medicare and Medicaid Services (CMS) website. We proceeded to use the number of patients as the indicator of the market share for the institutions. The data published by CMS do not directly report the patient number. However, each facility reports its number of respondents, which we use as the proxy for our utilization measure.

#### 2.4.3. Health Facility Indicators

The quality of healthcare facilities is measured with two indicators, patient readmission rate and patient satisfaction rate, for each institution within the county. The average values are obtained for each county. The readmission rate is defined by CMS as an admission to an acute care hospital within 30 days of discharge from the same or another acute care hospital for all causes, and thus the cause of the readmission does not need to be related to the cause of the initial hospitalization [32].

Patient satisfaction ratings were obtained from a survey conducted by the Hospital Consumer Assessment of Healthcare Providers and Systems (HCAHPS), a national, standardized survey of patients which asks about their experiences during a recent inpatient hospital stay [33]. Patients who stayed in the hospital were asked 27 questions, including experiences with nurses, doctors, environments, and general treatment. We used one specific measure, “Using any number from 0 to 10, where 0 is the worst hospital possible and 10 is the best hospital possible, what number would you use to rate this hospital during your stay?”, to capture the general patients’ satisfaction with the healthcare facilities.

#### 2.4.4. Hierarchical Condition Category (HCC) Score

The aspects of geographic variation have also long been considered phenomenal, including health status in terms of diagnosis intensity and cost variation [34]. In this study, the HCC score was employed as a proxy for county residents’ health conditions. It estimates how beneficiaries’ fee for service (FFS) spending will compare to the overall average for the entire Medicare population. Thus, it is a risk factor for the health spending of the Medicare population. The CMS-HCC model is normalized to 1.0. Beneficiaries were considered relatively healthy and, therefore, less costly with a risk score less than 1.0. Beneficiaries with scores greater than one are expected to have above average spending, and vice versa. In other words, a higher score implies poorer health since spending is higher. [35] The HCC is generally regarded as the best risk-adjustment model available and is used by CMS for both Medical Advantage plan and (in a modified form) Part D payment.

#### 2.4.5. Demographics, Geographics, and Other Control Variables

Variations in age, race and sex ratios across regions are driving factors for many diseases [36,37]. In addition to Black, Asian, and Hispanic, this study includes multiracial as an identity in the race category. A multicultural background may suggest a different perspective on adapting to new ideas about disease control. For infectious diseases, population density is presumed to be a critical factor since the ability to maintain social distancing has been widely emphasized in preventing disease. Thus, geographic factors are included in our models of estimation as control variables, including population density, county area, and travel time to work.

## 3. Data

A total of 3042 counties in the United States are the observation units in the study. Some counties are not included due to the unavailability of data, primarily because their populations are less than 500 and data are not collected for them. The outcome measures are extracted from USAFacts [38], which collects COVID-19 data from multiple sources, including the Centers for Disease Control and Prevention (CDC) and state- and local-level public health agencies. Quality measures for healthcare institutions in the United States are sourced from CMS [39] under the CAHPS^®^ (Consumer Assessment of Healthcare Providers and Systems) Healthcare Institution Survey, a national survey of family members or friends who cared for a patient who died in the care of a healthcare institution. Detailed descriptions of the quality measurement can be obtained from the CMS webpage [39].

The aforementioned socioeconomic, demographic, and county characteristics are compiled and published by the Census Bureau of the U.S. government [40]. Population, race ethnicity data, and sex ratios are obtained from the Population Estimates Program (PEP). Employment, education, income, transportation, and housing data are obtained from the American Community Survey (ACS). Uninsured population and disability data are obtained from the Current Population Survey (CPS), Annual Social and Economic Supplement (ASEC); State level—American Community Survey (ACS), one-year estimates; and County level—The Small Area Health Insurance Estimates (SAHIE) [41]. Geographic data are obtained from the Geography Division based on the TIGER/Geographic Identification Code Scheme (TIGER/GICS) computer file.

### 3.1. Empirical Strategies

#### 3.1.1. Quantile Regression (QR) Model

Since areas with different levels of infection severity may have different causes and risk factors, subdividing the analyses into different quantiles based on the population infection rate is of interest. Quantile regression (QR) analysis was proposed as an expansion of the least absolute deviation (LAD) [42]. QR has been used to detail the performance of explanatory variables under the influence of conditional medians. The benefit of QR estimation is that the models describe the performances of different quantile conditional distributions and, therefore, more comprehensively describe the characteristics of samples. This model is different from the OLS model, which only describes the mean marginal effects of the explanatory variables on the explained variables.

Based on the conventional descriptions of the QR study [42], we established a random variable cumulative distribution function, as shown in Equation (2).

(2) $$Py(y_{i}<y|x_{i})\;=\;F(y\;-\;x_{i}\;\beta |x_{i})\;=\tau ,\;\tau \;∊\;(0,\;1)$$

where *y_i_* represents the dependent variable vector for county i, and *x_i_* is the independent explanatory variable vector, including socioeconomics, demographics, health indicators, and other control characteristics of the counties. *β* is the regression coefficient vector obtained through an estimation satisfying Equation (1) and varies according to different quantiles *τ*. Therefore, *β*(*τ*) represents the regression coefficient vector under the effect of the *τ*th quartile.

We simplified Equation (1) into a basic cross-sectional data quantile regression model, as shown in Equation (3).

(3) $$Y_{\mathrm{it}}=\alpha_{i}+x^{\prime }\;\beta_{i}+\varepsilon_{i}\left(\tau \right)$$

where *ε_i_* *(τ)* represents the random error under quantile *τ* assuming E(*ε*_I_ (*τ*)*|x_i_*) = 0, and *α_i_* represents the area fixed effects (Koenker, 2004). The value of $\hat{\beta }\left(\tau \right)$ estimated by QR under fixed effects represents the marginal effects of different quantile explanatory variables on the explained variables when other explanatory variables *x_i_* were controlled.

The bootstrap method for sampling estimation was employed, and resampling was used to simulate the population distribution [43]. We also relaxed the assumption limit, which requires the conditional distribution of the errors to be homoscedastic [44]. Thus, a variance matrix estimation equation was obtained with consistency.

#### 3.1.2. Hierarchical Regression Model

In this research, the observation unit is a county in one of the 50 U.S. states, nested in ten regions based on the classifications of the Center for Medicare and Medicaid Services (CMS); thus, a model of two-level nested groups was constructed. This hierarchy is suitable for applying mixed-effects models, which are characterized as containing both fixed effects and random effects; the former are analogous to standard regression coefficients and are estimated directly, and the latter are not directly estimated but are summarized according to their estimated variances and covariances. Random effects may take the form of either random intercepts or random coefficients in the nested groups. Multilevel models, also known as hierarchical models, have been used extensively in diverse fields, ranging from the health and social sciences to econometrics. [45,46,47] Our regression models take the following form:

(4) $$Y_{i,s,\;r}=\beta_{0}+\beta_{1}X_{i,s,\;r}+\delta_{r}+\lambda_{s,r}+\varepsilon_{i,s,r}$$

where i, s, and r denote the county, state, and CMS regions, respectively. Y represents the variables of interest for investigation: spread control and the infection rate of COVID-19. X denotes independent variables, including socioeconomic, demographic, health indicators, and other control variables. The region and state error terms and residual are, respectively, δ~ [N(0, σ_δ_^2^))], *λ*_j_ ~[N(0, σ_*λ*_^2^))], and ε_ijt_ ~[N(0, σ_ε_^2^)].

#### 3.1.3. Random Forest Model

The regression coefficients derived from the abovementioned models are a measure of the association between a particular feature and the outcomes. We supplemented our analyses with a random forest machine learning algorithm, which produces computed feature importance values and provides information about the relative importance of each feature for predicting outcomes for the entire sample. The importance value of each feature in the models determines which variables were the most important for determining the speed of disease spread and severity of the infection condition. The STATA software package is employed for the prediction of the random forest model, in which variable importance is calculated by summing the improvement in the objective function obtained from the splitting criterion over all internal nodes of a tree and across all trees in the forest. The process is generated through the mean decrease Gini. The outcome variables and the regressors are identical to those described in the aforementioned models. Additional details of the statistical analysis and feature engineering are available in studies by Breiman (2001) and Zou and Schonlau (2019) [48,49].

## 4. Results

The characteristics of the areas with severe and mild infection incidents might be very different. Thus, we divided our observation units, the U.S. counties, into terciles based on the infection rate: mild, moderate, and severe. Table 1 and Table 2 show the summary statistics for the whole sample and the three groups. Mildly infected areas have a higher percentage of the white population, a lower percentage of the black population, a lower percentage of foreign-born people, a greater percentage of the older population, and a greater percentage of owner-occupied housing than severely infected areas. Regarding healthcare indicators, mildly infected areas have more concentrated healthcare institutions, institutions have lower readmission rates, and the population has fewer risk factors, as measured by general healthcare spending, than severely infected areas. All the differences are statistically significant at the 1% or 5% level.

Data are submitted to ordinary least square (OLS) and quantile regression methods to investigate whether relationships exist between those factors and the control of spread and the infection conditions of the disease. The results are presented in Table 3, Table 4, Table 5 and Table 6. For the spread of the disease, the risk factors that facilitate (have a negative effect on) disease spread include the female labor ratio, percentage of population under 18 years old, percentage of the population over 65 years of age, and HCC. All factors are statistically significant at a 1% level for at least two of the three quantile groups. Counties’ median housing value, land size, travel time to work, female population ratio, race mix, and HHI are protective factors that are positively related to the length of time to reach the 3% infection rate. Some factors exert opposite effects when measuring different quantiles of infection conditions, such as income and the percentage of the population with a college degree. The regression results for the analyses of infection rates are presented in Table 4. Income, broadband internet coverage, travel time to work, elderly population ratio, and college graduate ratio with negative effects on the infection rate are proactive factors, while employment ratio, population density, percentage of owner-occupied housing, the population ratio under 18 years, and percentage of uninsured individuals are positively related to the infection rate. Based on the R^2^ value of the results, the model of the severe tercile has a better fit than those of OLS and the other two terciles. Our next step is to include the state-fixed effects in the models to increase the precision of the estimate, and the results return a better fit of R^2^ and more variables with statistically significant coefficients, as presented in Table 5 and Table 6. Generally, the signs of the coefficients are consistent with those in the models without fixed effects. The differences are that the coefficients of the hospital readmission rate and income are no longer significant in the control of disease spread model, and the female labor population rate and broadband coverage are no longer significant in the infection rate estimation. More interestingly, the coefficients of the uninsured population change to negative at the 1% statistical significance level.

**Figure 3:**
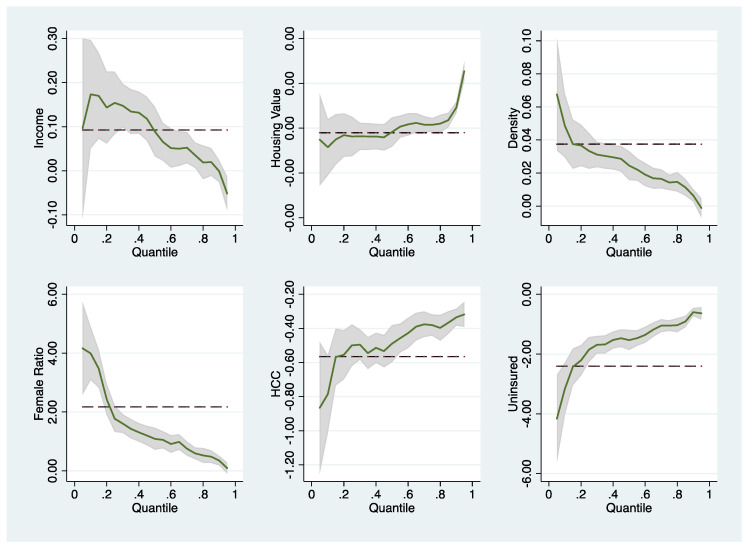
Effects of selected factors at different quantiles: Model of the control of COVID-19 spread.

**Figure 4:**
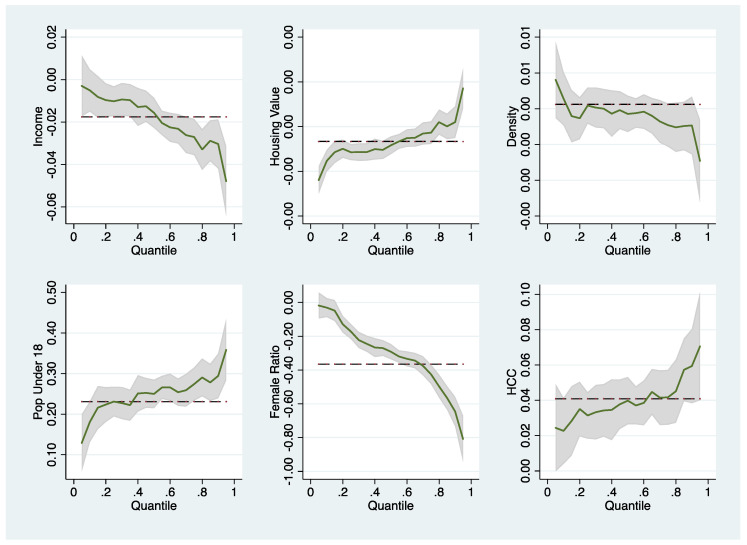
Effects of selected factors at different quantiles: Model of the COVID-19 infection rate.

Selected variables were submitted for quantile graphic presentation, as shown in Figure 3 and Figure 4, to obtain a clearer picture of the extent to which the effects of the covariates on the outcomes vary across infection severity levels. For disease spread, income, density, and female ratio exert a strong positive effect on the control of infection spread when the disease condition is mild. However, as the infection rates deteriorated, the effect vanished gradually. On the other hand, housing value, HCC, and uninsured ratio exert prominent negative effects on the control of disease spread when the infection condition is mild, and the effect diminishes as the quantile approaches 1 (most severe). The opposite effect to what we found in the spread control model was observed for the infection incidence rate. All the variables illustrated in Figure 4 exert moderate effects when counties have mild infection rates, and the effects intensify when infection conditions worsen.

**Figure 5:**
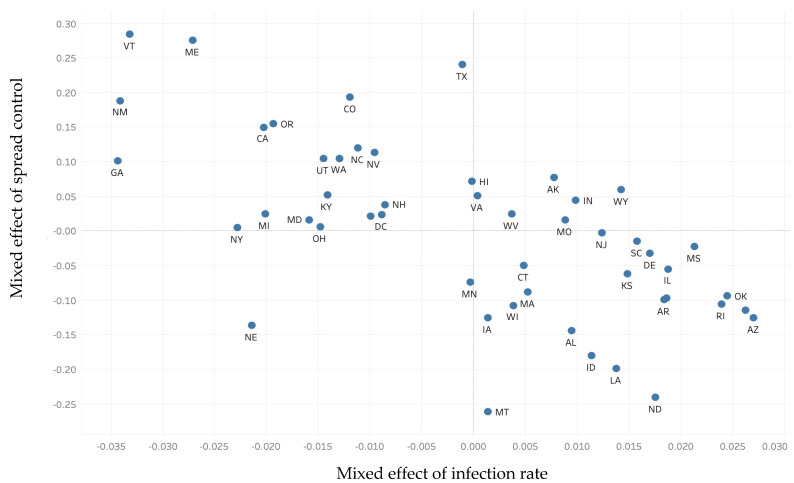
Random effects at the state level. Notes: The vertical axis indicates the effect extracted from the spread model, and the horizontal axis represents the effect extracted from the infection rate model. A higher value indicates better spread control, and a shift to the right indicates a higher infection rate. The second quadrant shows the states with better than average outcomes (slow spread and low infection rates); the fourth quadrant shows the states with worse than average outcomes (fast spread and high infection rates) in the first wave of disease statistics.

The data were further examined using a hierarchical regression analysis (mixed random and fixed effect model) to assess the robustness of the results. Similar results are obtained, as shown in Table 7. The state mixed effects for both outcome measurements are collected after the analyses, and the two-dimensional plot is shown in Figure 5, where the vertical axis represents the effects extracted from the spread model, and the horizontal axis represents the effect extracted from the infection rate model. The second quadrant shows the states with slow spread and low infection rates; the fourth quadrant shows the states with fast disease spread and high infection rates in the pre-Delta period of disease statistics. An apparent negative trend between the two outcome variables is observed, suggesting that better control of disease spread would lead to a lower infection rate.

**Figure 6:**
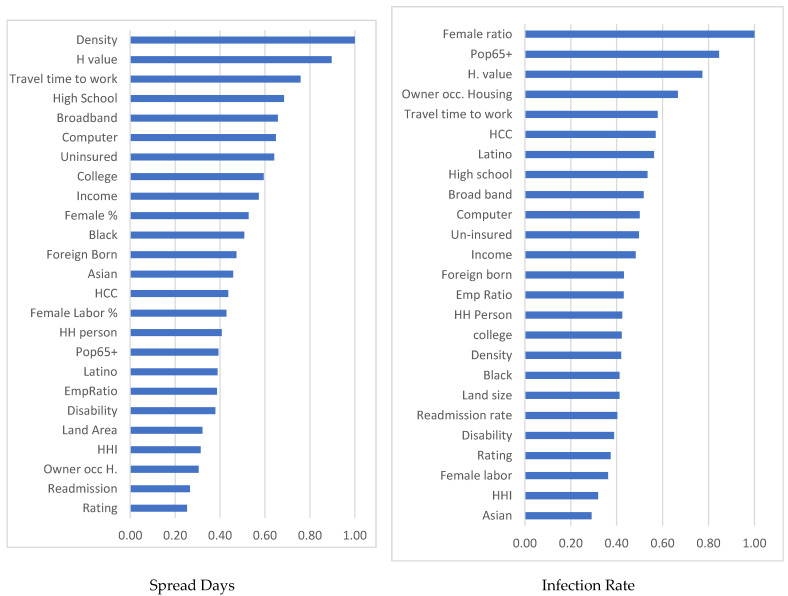
Random forest prediction for importance. Notes: The H value represents medium housing value; HH person represents number of people per household, EmpRatio represents the employment to population ratio, Rating indicates the average hospital rating.

Finally, random forest modeling is employed using the standard procedures designed by Breiman [44] and Frank et al. [50] to understand the relative importance of each explanatory variable. The prediction performances of the models are approximated using the out-of-bag (OOB) errors [51]. After a bootstrap of 1500 iterations, OOB errors of 0.027 and 0.204 are obtained for the models of infection rate and spread control, respectively. The results of the relative feature importance (FI) for the two outcome variables are shown in Figure 6, mainly confirming the findings of the regression analysis.

## 5. Discussion

At this time of unpredictable pandemic upticks due to evolving SARS-CoV-2 variants, studies are needed to provide insights into the risk factors determining the spread and the incidence rates of this contagious disease in a timely manner. This study investigates the key factors that influence the spread of COVID-19 and the variation in infection rates across the United States. During the outbreak of respiratory diseases when vaccines are not yet available, understanding the risk and preventive factors might help control the spread and gain more time for scientists to develop new vaccines and treatment methods. Results of these studies would help government authorities allocate medical resources, prepare for disease prevention, and plan strategically to achieve better management in disease control.

Intuitively, counties with better spread control would have a lower infection rate. Using the U.S. county data and various statistical models, our results indicate that most factors in the two models exert consistent effects, indicating that if they are protective factors for the control of disease spread (have positive effects), they also tend to be protective factors (have a negative effect) on COVID-19 infection rates. Analogously, if the factors exert negative effects on the control of disease spread or the risk factors, they tend to positively affect the infection rate. Our results reveal that for the spread control model, the effects of risk and preventative factors are more prominent in the counties with mild infection conditions than in the counties with severe infection conditions, while the opposite results are observed for the models of infection rate, i.e., they have stronger effects on severely infected counties than on mildly infected counties. For example, the female population ratio is a preventive factor for the control of disease spread. As the female ratio increases by 1%, the spread is delayed by 1.20% when the infection is mild. However, the delay is only 0.5% when infection is in the moderate tercile, and no delay (insignificant) is observed in the severe tercile. Another example is the effect of median income on the infection rate. As income increases by 1%, the infection rate decreases by 0.05% (*p* < 0.05) for the severely infected counties. However, the effect is only 0.005% and 0.008% for the mildly and moderately infected counties, respectively, and the result is not statistically significant in the latter cases.

The risk factors are generally the employment to population ratio, young population (18 years or younger) ratio, female labor ratio, and residents’ general health risk measured using the HCC. The protective factors include median housing value, broadband internet coverage, land size, travel time to work, female population ratio, multiracial ratio, HHI, median household income, and land area. These factors have different levels of effect and various levels of statistical significance when measured using spread control models or infection rate models. In both models, the factors showing consistent significance levels include housing value, land size, travel time to work, female population ratio, and multiracial ratio as preventive factors and employment ratio, female labor ratio, young population ratio, and HCC as risk factors.

Some factors exert opposite effects on the spread control and infection rate models. For example, more densely populated areas tend to have higher infection rates. However, population density is a protective factor when disease conditions are mild or moderate in the disease spread control model. This discrepancy is probably because population density captures the characteristics of urbanization of the county. The percentage of the uninsured population also exerts the opposite effect; it is a risk factor for the control of disease spread but a preventive factor for the infection rate. This difference is probably because the uninsured group is usually younger and healthier, and thus, the infection rate is lower. Furthermore, this population might not think COVID-19 would cause too much harm and thus did not maintain precautions as a habit; thus, spread control was negatively affected.

Studying the nature of these factors suggests that personal hygiene may play an important role in promoting disease prevention. Different cohorts may share the characteristics of ease of adaptation or openness of attitudes toward new habits under certain circumstances. For example, a multiracial cohort and people residing in more urbanized areas might find it more acceptable to adopt new habits of more frequent handwashing, mask wearing, and social distancing. A higher elderly ratio is associated with less severe infection, possibly because the elderly, which comprise the high-risk group, would take special precautions and adopt new habits to prevent them from contracting the disease.

Another interesting finding is about the female role in society. A greater female ratio helps delay disease spread and lower the infection rate. However, greater female labor force participation exerts the opposite effect, implying that females devoting time to the work force do not spend the time necessary to ensure sanitary conditions for their families and increase the infection risk in their communities. In summary, the study results imply that taking precautions in personal hygiene is important in both spread control and decreasing the infection incidence rate, as manifested by special cohort groups who might share certain characteristics for high vigilance in personal hygiene. However, when the general disease condition continues to worsen, reaching the higher quantile in infection rates, these protective factors play less important roles in preventing the disease.

Finally, well-established healthcare institutions with greater market power are significantly protective for slowing disease spread, implying that competitiveness is a less ideal market structure in the healthcare industry. Although the protective effect vanishes when the infection rate becomes severe, reputation and quality of care are better served in an imperfectly competitive setting.

The feature importance (FI) values for the random forest models generally confirm the findings of the regression analysis. Population density, female population ratio, and travel time to work are the top three factors determining spread control. The female population ratio, elderly ratio, and housing value are the top factors determining the infection rate. People with a nicely comfortable home environment exhibit a greater tendency to stay home and reduce their interactions with people outside the family, which in turn reduces the probability of contracting COVID-19. The only factor that exhibits a difference in determining power between the random forest and the regression model is the HHI. In the random forest model, the HHI is located at the bottom as the next to the least important factor, while it appears to be one of the few significant explanatory variables in the quantile regression and mixed effect models. This similar discrepancy in the results appears in the existing studies and is probably because random forest models assign greater weight to prediction accuracy and the magnitudes of the coefficients instead of the causal relationship and the statistical significance of individual regressors [52]. This finding is noted as a limitation in the interpretability of this research.

## 6. Conclusions

Effectively containing the spread of infectious disease is essential in public health considerations, especially when vaccines and efficacious cures for the diseases are not yet available. In this study, we employ three popular and newly developed models to investigate the COVID-19 pandemic condition before the introduction of the vaccines, including quantile regression, hierarchical mixed effect model, and random forest models. Notably, both protective and risk factors for COVID-19 are incorporated as predictors. Our results suggest that the protective factors that slow disease spread and lower infection rates include land size, housing value, travel time to work, female ratio, HHI, and percentage of the population who identify themselves with more than one race (multiracial). Some of these protective factors are related to the ease of maintaining social distancing, while others may be linked to cohort characteristics for their attitudes toward adopting new habits that might be beneficial for disease prevention, such as the habits of maintaining personal hygiene, mask-wearing, and handwashing. Populations with more females and multiracial cohorts seem more adaptable to taking precautions with personal hygiene. Healthcare facilities with higher ratings that face less competition also play a more important role in controlling disease spread and lowering infection rates than facilities facing fierce competition. However, most of the protective factors only exert a significant effect when disease conditions are mild or moderate in the counties. When the disease condition worsens, the effects of protective factors diminish. On the other hand, risk factors, such as employment ratio, female labor ratio, and HCC, exert more prominent effects when the disease condition is aggravated.

The implications of the risk factors for our study are described below. First, bustling business interactions facilitate the spread of viruses. Second, more females in the labor market aggravate the disease condition. Females usually play the primary role in running the household. If they devote their time to the labor market, they spend less time and effort maintaining sanitary conditions for their families. Third, the health risk indicator of a county, the HCC, directly exerts a significant positive effect on disease severity.

Our study also reveals some other interesting findings. Although the elderly might be frail and vulnerable, the elderly ratio is not associated with a higher infection rate, probably because this population is more careful about maintaining social distancing and practicing personal hygiene because they know that they are at high risk once infected. The uninsured population represents a younger, active, and healthier group of people who accelerate disease spread, but in general, the overall infection rate in an area is not particularly worsened when a highly uninsured population is present.

Continuing efforts to maintain personal hygiene, social distancing, and mask wearing are crucial for controlling disease spread. These measures are particularly effective when the infection condition is not serious, as indicated in the low quantile of infection rates in this study. When the infection condition continues to deteriorate, these protective factors lose their effect, and the risk factors become more powerful in aggravating the situation.

This study provides insight into controlling contagious disease spread and the infection rate in terms of socioeconomics, demographics, and indicators of regional healthcare facilities. The findings ascertain the importance of personal precautions, broadband internet coverage, and large-scale healthcare facilities. Suggestions for future studies include continuous efforts to monitor pandemic conditions for ever-emerging variants and assess the relationships between the unvaccinated rate, hospitalization rate, death rate, and demographics and socioeconomic indicators. Refined statistical models and machine-learning algorithms should also be adopted for greater precision of predictions or better interpretability of artificial intelligence models, such as Shapley Additive Explanations (SHAP) [53].

## Figures and Tables

**Table 1:** Summary statistics stratified by infection rate.

	All	Mild (1)	Medium (2)	Severe (3)	Diff. *p* Value Btw (1) & (3)
Spread days	213.6	219.8	223.2	197.9	0.000 ***
	(60.84)	(62.23)	(66.22)	(49.93)	
County rate	0.132	0.09	0.13	0.16	0.000 ***
	(0.04)	(0.02)	(0.01)	(0.02)	
County cases	19.04	4.12	28.96	24.13	0.000 ***
(Max.)	(65.00)	(31.59)	(71.10)	(79.27)	
Demographic					
Female %	0.499	0.498	0.501	0.499	0.331
	(0.02)	(0.02)	(0.02)	(0.02)	
White %	0.843	0.867	0.821	0.843	0.001 ***
	(0.16)	(0.15)	(0.17)	(0.17)	
Black %	0.0960	0.0769	0.118	0.0934	0.003 ***
	(0.146)	(0.132)	(0.157)	(0.145)	
Asian %	0.0157	0.0149	0.0180	0.0143	0.411
	(0.0283)	(0.0312)	(0.0312)	(0.0214)	
Latino %	0.0975	0.0903	0.101	0.101	0.055 *
	(0.139)	(0.137)	(0.135)	(0.144)	
Foreign born %	0.0478	0.0428	0.0515	0.0490	0.023 **
	(0.0574)	(0.0538)	(0.0578)	(0.0601)	
65 years+	0.196	0.201	0.195	0.192	0.002 **
	(0.0464)	(0.0473)	(0.0486)	(0.0428)	
College education	0.219	0.217	0.228	0.214	0.412
	(0.0957)	(0.0893)	(0.105)	(0.0912)	
Persons in household	2.514	2.491	2.538	2.514	0.111
	(0.264)	(0.264)	(0.258)	(0.267)	
Economics					
Employment%	0.281	0.277	0.275	0.291	0.015 **
	(0.127)	(0.125)	(0.127)	(0.129)	
Female labor %	0.539	0.539	0.537	0.542	0.433
	(0.0699)	(0.0682)	(0.0708)	(0.0705)	
Income (1000)	53.44	53.25	54.21	52.83	0.323
	(14.13)	(13.10)	(15.64)	(13.52)	
Owner-occupied housing	0.716	0.724	0.711	0.714	0.028 **
	(0.0817)	(0.0828)	(0.0832)	(0.0786)	
Broadband internet	0.754	0.756	0.758	0.750	0.071 *
	(0.09)	(0.084)	(0.09)	(0.09)	
Travel time to work	24.05	23.79	25.01	23.36	0.056 *
(min)	(5.736)	(5.721)	(5.944)	(5.414)	
Social					
Disability %	0.111	0.111	0.113	0.110	0.487
	(0.04)	(0.04)	(0.04)	(0.04)	
No insurance %	0.119	0.115	0.121	0.120	0.028 *
	(0.05)	(0.05)	(0.051)	(0.051)	
Poverty %	0.145	0.142	0.146	0.146	0.056 *
	(0.056)	(0.054)	(0.059)	(0.06)	
Pop. Density	266.1	170.9	319.1	308.6	0.088 *
	(1752.6)	(659.0)	(1539.1)	(2525.0)	

Notes: Standard deviations are shown in parentheses. County cases and county rate represent the peak figures of the pre-Delta period, which decreased in approximately February 2021. Each county peaked on different dates. * *p* < 0.10, ** *p* < 0.05, *** *p* < 0.010.

**Table 2:** Summary Statistics—continued.

	All	Tercile (1)	Tercile (2)	Tercile (3)	Diff. *p* Value Btw (1) & (3)
Healthcare Characteristics					
HHI	8322.1	8458.5	8221.8	8284.9	0.012 ***
	(2438.0)	(2277.7)	(2498.7)	(2526.5)	
Rating	88.82	88.94	88.64	88.88	0.531
	(2.213)	(2.219)	(2.100)	(2.306)	
HCC	0.964	0.952	0.967	0.972	0.000 ***
	(0.104)	(0.102)	(0.105)	(0.104)	
Readmission Rate	16.87	16.72	16.89	16.99	0.043 **
	(3.033)	(2.962)	(2.992)	(3.139)	
N	3042	1015	1014	1013	

Notes: Standard deviations are shown in parentheses. HHI represents the Hirfindahl–Hirschman Index. Rating represents the average patient satisfaction rating toward hospitals in the county, and HCC represents the hierarchical condition category. ** *p* < 0.05, *** *p* < 0.010.

**Table 3:** Determinants of COVID-19 Spread.

	OLS	Tercile (1)	Tercile (2)	Tercile (3)
Socioeconomics				
Income	0.096	0.056	0.145 ***	−0.348 **
	(1.50)	(1.45)	(3.72)	(−2.24)
Employment (%)	−0.103	−0.099 *	−0.102 **	0.170
	(−1.43)	(−1.60)	(−2.18)	(1.18)
Female Labor (%)	−0.607 ***	−0.544 ***	−0.579 ***	−0.054
	(−3.90)	(−3.65)	(−5.68)	(−0.14)
Housing Value	0.059 **	0.069 ***	0.046 **	0.276 ***
	(2.09)	(2.85)	(2.16)	(3.60)
Broadband (%)	0.155	0.305 ***	0.166 *	0.469
	(1.17)	(3.24)	(1.86)	(1.36)
Owner-occupied Housing (%)	0.079	−0.057	−0.176	−0.309
	(0.32)	(−0.37)	(−0.82)	(−0.67)
Land Size	0.083 ***	0.060 ***	0.031 ***	0.044 **
	(7.47)	(7.22)	(4.43)	(1.93)
Density	0.081 ***	0.085 ***	0.046 ***	−0.034
	(8.30)	(8.59)	(8.46)	(−1.33)
Travel Time	0.176 ***	0.176 ***	0.132 ***	0.217 **
	(4.01)	(5.30)	(4.44)	(2.03)
Demographics				
Asian (%)	−0.944 ***	−0.528 **	−0.309 ***	−1.945 **
	(−3.00)	(−2.00)	(−2.59)	(−2.56)
Female (%)	2.634 ***	2.104 ***	1.333 ***	−0.390
	(5.94)	(4.37)	(4.28)	(−0.28)
Black (%)	−0.397 ***	−0.491 ***	−0.326 ***	−0.063
	(−8.58)	(−10.86)	(−7.04)	(−0.39)
Latino (%)	0.006	0.081	0.125 *	0.010
	(0.09)	(1.44)	(1.87)	(0.09)
Mixed Race (%)	3.551 ***	2.962 ***	2.721 ***	4.167 ***
	(9.10)	(9.84)	(9.51)	(3.10)
Under 18 Years (%)	−2.324 ***	−1.618 ***	−1.773 ***	−1.660 **
	(−6.73)	(−4.84)	(−6.20)	(−1.93)
Over 65 Years (%)	−0.793 ***	−0.388	−0.336	0.368
	(−2.88)	(−1.28)	(−1.11)	(0.38)
High School Education(%)	0.666 ***	0.776 ***	0.398 **	−0.113
	(2.75)	(3.28)	(2.30)	(−0.28)
College Education (%)	−0.163	−0.420 ***	−0.246 ***	1.162 ***
	(−1.32)	(−4.43)	(−3.02)	(3.39)
Health Indicators				
Uninsured (%)	−0.377 **	−0.833 ***	−0.260 **	0.307
	(−2.54)	(−5.60)	(−2.01)	(0.96)
Rating	0.006 **	0.002	−0.002	0.002
	(2.44)	(0.69)	(−1.30)	(0.34)
Readmission Rate	0.005 **	0.003	0.004 **	0.012 *
	(1.91)	(1.32)	(1.98)	(1.82)
HCC	−0.275 ***	−0.241 ***	−0.333 ***	−0.217
	(−2.70)	(−3.19)	(−6.67)	(−0.86)
HHI	0.084 ***	0.068 ***	0.035 ***	0.038
	(5.33)	(4.37)	(3.55)	(0.65)
R-sq	0.23	0.17	0.19	0.03

Notes: Standard deviations are shown in parentheses. * *p* < 0.10, ** *p* < 0.05, and *** *p* < 0.010. Income, medium housing value, land size, and travel time to work are presented in natural logarithm.

**Table 4:** Determinants of the COVID-19 Infection Rate.

	OLS	Tercile (1)	Tercile (2)	Tercile (3)
Socioeconomics				
Income	−0.024 ***	−0.015 *	−0.008	−0.036 *
	(−3.28)	(−1.74)	(−0.88)	(−1.76)
Employment (%)	0.042 ***	0.040 ***	0.040 ***	−0.010
	(4.94)	(4.65)	(4.75)	(−0.29)
Female Labor (%)	−0.001	0.004	−0.020	−0.028
	(−0.07)	(0.16)	(−1.03)	(−0.42)
Housing Value	−0.000	0.000	−0.000	0.000
	(−0.29)	(0.22)	(−0.34)	(0.43)
Broadband (%)	−0.022	−0.043 ***	−0.028 **	−0.070 **
	(−1.49)	(−3.50)	(−2.27)	(−1.98)
Owner-occupied Housing (%)	0.091 ***	0.114 ***	0.076 **	0.117 *
	(4.16)	(3.60)	(2.53)	(1.82)
Land Size	0.001	0.003 **	0.000	−0.007 *
	(0.71)	(2.31)	(0.35)	(−1.68)
Density	0.003 ***	0.005 ***	0.002 *	−0.004
	(2.90)	(3.75)	(1.70)	(−1.45)
Travel Time	−0.018 ***	−0.021 ***	−0.027 ***	−0.062 ***
	(−4.00)	(−3.53)	(−4.19)	(−3.20)
Demographics				
Asian (%)	−0.053 **	−0.041	−0.082 ***	0.042
	(−2.03)	(−1.54)	(−3.42)	(0.25)
Female (%)	−0.256 ***	−0.128 **	−0.238 ***	−0.484 ***
	(−4.38)	(−2.14)	(−4.44)	(−4.25)
Black (%)	−0.013 **	−0.011 **	−0.004	−0.014
	(−2.48)	(−2.29)	(−0.49)	(−0.62)
Latino (%)	−0.012	−0.015 **	−0.030 ***	0.055
	(−1.53)	(−1.93)	(−3.66)	(1.39)
Mixed Race (%)	−0.245 ***	−0.247 **	−0.216 ***	−0.362 **
	(−5.41)	(−2.54)	(−4.09)	(−2.42)
Under 18 Years (%)	0.179 ***	0.196 ***	0.132 ***	−0.033
	(5.41)	(5.49)	(3.30)	(−0.27)
Over 65 Years (%)	−0.148 ***	−0.177 ***	−0.160 ***	−0.284 ***
	(−5.02)	(−5.15)	(−4.47)	(−3.10)
High School Education (%)	−0.035	−0.022	−0.054 *	−0.046
	(−1.34)	(−0.85)	(−1.84)	(−0.46)
College Education (%)	−0.045 ***	−0.054 ***	−0.051 ***	0.055
	(−3.57)	(−3.41)	(−3.07)	(0.75)
Health Indicators				
Uninsured (%)	0.057 ***	0.086 ***	0.105 ***	0.110 *
	(3.51)	(4.72)	(5.15)	(1.69)
Rating	−0.000	0.000	−0.001 ***	−0.001
	(−1.32)	(0.34)	(−2.67)	(−1.24)
Readmission Rate	−0.000	−0.000	0.000	−0.000
	(−0.12)	(−0.57)	(0.29)	(−0.26)
HCC	0.046 ***	0.042 ***	0.057 ***	0.095 ***
	(4.13)	(3.90)	(4.45)	(2.62)
HHI	−0.002	−0.001	−0.003	0.004
	(−0.99)	(−0.31)	(−1.51)	(0.73)
R-sq	0.30	0.18	0.18	0.35

Notes: Standard deviations are shown in parentheses. * *p* < 0.10, ** *p* < 0.05, and *** *p* < 0.010. Income, medium housing value, land size, and travel time to work are presented in natural logarithm.

**Table 5:** Spread Days with Fixed Effects.

	OLS	Tercile (1)	Tercile (2)	Tercile (3)
Socioeconomics				
Income	0.039	−0.005	0.034	−0.079
	(0.53)	(−0.09)	(0.88)	(−1.13)
Employment (%)	−0.124 *	−0.114 ***	−0.060	0.008
	(−1.74)	(−2.62)	(−1.47)	(0.09)
Female Labor (%)	−0.216	−0.194	−0.252 **	−0.248 *
	(−1.38)	(−1.17)	(−2.47)	(−1.72)
Housing Value	0.019	0.108 ***	0.077 ***	0.104 ***
	(0.51)	(3.41)	(5.33)	(3.97)
Broadband (%)	0.042	0.214 *	0.035	−0.089
	(0.33)	(1.84)	(0.49)	(−0.75)
Owner-occupied Housing (%)	0.085	−0.049	0.030	0.181
	(0.33)	(−0.30)	(0.24)	(0.81)
Land Size	0.074 ***	0.063 ***	0.032 ***	−0.014
	(6.30)	(6.72)	(4.83)	(−0.61)
Density	0.073 ***	0.057 ***	0.033 ***	−0.011
	(6.89)	(9.39)	(4.58)	(−1.08)
Travel Time	0.123 ***	0.131 ***	0.078 ***	0.133 ***
to Work	(2.97)	(4.04)	(4.48)	(2.70)
Demographics				
Asian (%)	−1.137 ***	−0.455	−0.233 *	−0.207
	(−3.41)	(−1.04)	(−1.83)	(−0.49)
Female (%)	1.831 ***	1.196 **	0.490 *	−0.348
	(4.13)	(2.32)	(1.63)	(−0.44)
Black (%)	−0.389 ***	−0.371 ***	−0.164 ***	−0.103
	(−6.57)	(−6.38)	(−2.80)	(−1.32)
Latino (%)	−0.465 ***	−0.378 ***	−0.292 ***	−0.096
	(−4.77)	(−3.89)	(−4.17)	(−1.43)
Mixed Race (%)	2.723 ***	2.752 ***	2.408 ***	4.069 ***
	(3.82)	(5.95)	(3.99)	(5.24)
Under 18 Years (%)	−0.750 **	−0.329	−0.389 *	0.303
	(−2.11)	(−1.31)	(−1.74)	(0.50)
Over 65 Years (%)	−0.204	−0.063	0.064	0.381
	(−0.68)	(−0.28)	(0.45)	(0.89)
High School Education (%)	0.624 **	0.580 **	0.518 ***	0.029
	(2.42)	(2.55)	(3.06)	(0.14)
College Education (%)	0.011	−0.320 ***	−0.169 **	0.484 **
	(0.09)	(−3.02)	(−2.39)	(2.18)
Health Indicators				
Uninsured (%)	−0.995 ***	−0.781 **	−0.351 *	−0.516 *
	(−2.81)	(−2.46)	(−1.61)	(−1.77)
Rating	0.005 **	0.003 *	−0.001	−0.001
	(2.10)	(1.76)	(−0.38)	(−0.47)
Readmission Rate	0.002	0.000	0.001	0.000
	(0.75)	(0.06)	(0.80)	(0.17)
HCC	−0.203 *	−0.140	−0.163 ***	−0.059
	(−1.88)	(−1.44)	(−2.78)	(−0.52)
HHI	0.045 ***	0.025 ***	0.017 **	−0.021
	(3.02)	(3.02)	(1.92)	(−0.95)
R-sq	0.49	0.37	0.36	0.64
Observations	3042	3042	3042	3042

Notes: Standard deviations are shown in parentheses. * *p* < 0.10, ** *p* < 0.05, and *** *p* < 0.010. Income, medium housing value, land size, and travel time to work are presented in natural logarithm.

**Table 6:** Infection Rate with Fixed Effects.

	OLS	Tercile (1)	Tercile (2)	Tercile (3)
Socioeconomics				
Income	−0.019 ***	−0.005	−0.008	−0.046 **
	(−2.59)	(−0.60)	(−1.16)	(−2.18)
Employment (%)	0.031 ***	0.030 ***	0.031 ***	0.018
	(4.23)	(5.26)	(4.14)	(0.78)
Female Labor (%)	0.039 **	0.030 *	0.034 **	0.047
	(2.47)	(1.69)	(2.01)	(1.07)
Housing Value	0.000	0.000	0.000	0.000
	(1.25)	(0.01)	(0.45)	(1.31)
Broadband (%)	−0.003	−0.014	−0.012	−0.012
	(−0.24)	(−1.46)	(−0.95)	(−0.33)
Owner-occupied Housing (%)	0.016	0.006	−0.008	0.048
	(0.74)	(0.27)	(−0.36)	(0.83)
Land Size	0.001	0.003 ***	−0.000	−0.007 **
	(0.93)	(2.81)	(−0.42)	(−2.25)
Density	0.004 ***	0.005 ***	0.003 ***	−0.005
	(4.55)	(5.15)	(3.57)	(−1.45)
Travel Time	−0.018 ***	−0.013 ***	−0.018 ***	−0.028 **
to Work	(−5.07)	(−3.99)	(−4.29)	(−2.05)
Demographics				
Asian (%)	−0.050 **	−0.044	−0.076 **	0.121
	(−1.94)	(−1.46)	(−2.50)	(0.68)
Female (%)	−0.240 ***	−0.091 **	−0.250 ***	−0.606 ***
	(−4.41)	(−2.01)	(−8.17)	(−5.60)
Black (%)	−0.032 ***	−0.032 ***	−0.030 ***	−0.024
	(−5.68)	(−5.45)	(−6.01)	(−1.51)
Latino (%)	0.022 **	0.019	0.015 *	0.104 ***
	(2.28)	(1.33)	(1.60)	(3.48)
Mixed Race (%)	−0.276 ***	−0.234 **	−0.221 **	0.122
	(−4.00)	(−2.40)	(−2.46)	(0.49)
Under 18 Years (%)	0.087 ***	0.106 ***	0.118 ***	0.102
	(2.86)	(3.23)	(3.18)	(1.06)
Over 65 Years (%)	−0.048 *	−0.059 **	−0.037	0.020
	(−1.75)	(−2.03)	(−1.13)	(0.22)
High School Education (%)	−0.052 **	−0.061 **	−0.098 ***	−0.147 *
	(−1.99)	(−2.41)	(−5.45)	(−1.84)
College Education (%)	−0.079 ***	−0.076 ***	−0.065 ***	−0.038
	(−6.96)	(−4.93)	(−3.83)	(−0.88)
Health Indicators				
Uninsured (%)	−0.134 ***	−0.125 ***	−0.132 ***	−0.354 ***
	(−3.89)	(−3.31)	(−4.08)	(−2.99)
Rating	0.000	−0.000	0.000	−0.001
	(0.08)	(−0.64)	(0.17)	(−1.02)
Readmission Rate	0.000	0.000	0.000	0.000
	(0.04)	(0.32)	(0.04)	(0.53)
HCC	0.037 ***	0.029 ***	0.028 **	0.095 **
	(3.53)	(2.59)	(2.47)	(2.27)
HHI	0.001	0.003 **	−0.001	−0.000
	(0.52)	(2.26)	(−0.53)	(−0.09)
R-sq	0.60	0.43	0.41	0.53
Observations	3042	3042	3042	3042

Notes: Standard deviations are shown in parentheses. * *p* < 0.10, ** *p* < 0.05, and *** *p* < 0.010. Income, medium housing value, land size, and travel time to work are presented in natural logarithm.

**Table 7:** Mixed Effect Model.

	Spread Days	Infection Rate
Socioeconomics		
Income	0.027	−0.018 ***
	(0.51)	(−3.80)
Employment (%)	−0.120 **	0.031 ***
	(−2.18)	(6.07)
Female Labor (%)	−0.224 *	0.038 ***
	(−1.69)	(3.05)
Housing Value	0.030	0.000
	(1.08)	(1.42)
Broadband (%)	0.054	−0.003
	(0.54)	(−0.35)
Owner-occupied Housing (%)	0.105	0.018
	(0.60)	(1.08)
Land Size	0.077 ***	0.001
	(8.34)	(1.02)
Density	0.072 ***	0.004 ***
	(8.85)	(5.98)
Travel Time to Work	0.126 ***	−0.018 ***
	(3.96)	(−6.03)
Demographics		
Asian (%)	−1.058 ***	−0.050 **
	(−4.36)	(−2.16)
Female (%)	1.869 ***	−0.241 ***
	(6.38)	(−8.82)
Black (%)	−0.389 ***	−0.031 ***
	(−7.45)	(−6.35)
Latino (%)	−0.430 ***	0.020 ***
	(−6.88)	(3.39)
Mixed Race (%)	3.203 ***	−0.278 ***
	(6.20)	(−5.46)
Under 18 Years (%)	−0.840 ***	0.088 ***
	(−3.03)	(3.44)
Over 65 Years (%)	−0.224	−0.050 **
	(−0.96)	(−2.28)
High School Education (%)	0.620 ***	−0.050 ***
	(3.64)	(−3.10)
College Education (%)	−0.014	−0.078 ***
	(−0.13)	(−7.73)
HH Person	−0.096 *	−0.003
	(−1.95)	(−0.048)
Health Indicators		
Uninsured (%)	−1.011 ***	−0.122 ***
	(−4.14)	(−5.19)
Rating	0.005 **	0.000
	(1.92)	(0.11)
Readmission Rate	0.002	0.000
	(1.08)	(0.05)
HCC	−0.213 ***	0.038 ***
	(−2.66)	(5.23)
HHI	0.042	0.001
	(1.38)	(0.50)
AIC	36,620.066	−9713.874
BIC	36,764.537	−9569.396
chi^2^	307.860	69.530
*p*	0.000	0.000

Notes: Standard deviations are shown in parentheses. * *p* < 0.10, ** *p* < 0.05, and *** *p* < 0.010. Income, medium housing value, land size, and travel time to work are presented in natural logarithm.

## Data Availability

Data available on request from the corresponding author.

## References

[1] Farmer P. (1996). Social inequalities and emerging infectious diseases. Emerg. Infect. Dis..

[2] Uscher-Pines L., Duggan P.S., Garoon J.P., Karron R.A., Faden R.R. (2007). Social justice and disadvantaged groups. Hastings Cent. Rep..

[3] O’Sullivan T., Bourgoin M. (2010). Vulnerability in an Influenza Pandemic: Looking beyond Medical Risk.

[4] WHO (2008). Closing the Gap in a Generation: Health Equity through Action on the Social Determinants of Health.

[5] Quinn S.C., Kumar S. (2014). Health inequalities and infectious disease epidemics: A challenge for global health security. Biosecurity Bioterrorism Biodefense Strategy Pract. Sci..

[6] Woolf S.H., Aron L.Y., Dubay L., Simon S.M., Zimmerman E., Luk K. (2015). How Are Income and Wealth Linked to Health and Longevity?.

[7] Chetty R., Stepner M., Abraham S., Lin S., Scuderi B., Turner N., Bergeron A., Cutler D. (2016). The association between income and life expectancy in the United States, 2001–2014. JAMA.

[8] Blumenshine P., Reingold A., Egerter S., Mockenhaupt R., Braveman P., Marks J. (2008). Pandemic influenza planning in the United States from a health disparities perspective. Emerg. Infect. Dis..

[9] Diderichsen F., Evans T., Whitehead M. (2001). The Social Basis of Disparities in Health: Challenging Inequities in Health: From Ethics to Action.

[10] Biggerstaff M., Jhung M.A., Reed C., Fry A.M., Balluz L., Finelli L. (2014). Influenza-like illness, the time to seek healthcare, and influenza antiviral receipt during the 2010–2011 influenza season—United States. J. Infect. Dis..

[11] Tam K., Yousey-Hindes K., Hadler J.L. (2014). Influenza-related hospitalization of adults associated with low census tract socioeconomic status and female sex in New Haven County, Connecticut, 2007–2011. Influenza Other Respir. Viruses.

[12] Leon K., McDonald M.C., Moore B., Rust G. (2009). Disparities in influenza treatment among disabled Medicaid patients in Georgia. Am. J. Public Health.

[13] Dickman S.L., Himmelstein D.U., Woolhandler S. (2017). America: Equity and Equality in Health 1, Inequality and the health-care system in the USA. Lancet.

[14] Rae M., Levitt L., Claxton G., Cox C., Long M., Damico M. (2015). Patient Cost-Sharing in Marketplace Plans, 2016.

[15] Mellor J.M., Milyo J. (2001). Reexamining the Evidence of an Ecological Association between Income Inequality and Health. J. Health Politics Policy Law.

[16] Mellor J.M., Milyo J. (2002). Income Inequality and Individual Health: Evidence from the Current Population Survey. J. Human Res..

[17] Baciu A., Negussie Y., Geller A., Weinstein J.N., National Academies of Sciences, Engineering, and Medicine, Health and Medicine Division, Board on Population Health and Public Health Practice, Committee on Community-Based Solutions to Promote Health Equity in the United States (2017). Communities in Action: Pathways to Health Equity; The State of Health Disparities in the United States.

[18] HHS (2014). Infant Mortality Disparities Fact Sheets. http://minorityhealth.hhs.gov/omh/content.aspx?ID=6907&lvl=3&lvlID=8.

[19] NCHS (National Center for Health Statistics) (2016). Health, United States, 2015: With Special Feature on Racial and Ethnic Health Disparities.

[20] HHS (2016). Heart Disease and African Americans. http://minorityhealth.hhs.gov/omh/browse.aspx?lvl=4&lvlid=19.

[21] U.S Department of Health and Human Services Office of Minority Health, “Cancer and African Americans”. https://minorityhealth.hhs.gov/omh/browse.aspx?lvl=4&lvlid=16.

[22] Grossman M. (1972). On the Concept of Health Capital and the Demand for Health. J. Polit. Econ..

[23] Grossman M., Terleckyj N. (1976). The Correlation between Health and Schooling. Household Production and Consumption.

[24] Grossman M., Culyer A.J., Newhouse J.P. (2000). The Human Capital Model. Handbook of Health Economics.

[25] Fuchs V.R., Rogers D.E., Ginzberg E. (1993). Poverty and Health: Asking the Right Questions. Medical Care and the Health of the Poor.

[26] Arendt J.N. (2005). Does education cause better health? A panel data analysis using school reforms for identification. Economics of Education Review.

[27] Newhouse J.P., Friedlander L.J. (1980). The Relationship between Medical Resources and Measures of Health: Some Additional Evidence. J. Hum. Res..

[28] Ruhm C.J. (2000). Are Recessions Good for Your Health?. Q. J. Econ..

[29] Deaton A., Lubotsky D. (2003). Mortality, inequality and race in American cities and states. Soc. Sci. Med..

[30] (2006). Checklist for Pandemic Influenza Preparedness and Response Plans. The Bellagio Meeting on Social Justice and Influenza. http://www.bioethicsinstitute.org/wp-content/uploads/2012/12/Influenza-Checklist-English1.pdf.

[31] Deaton A. (2003). Health, Inequality, and Economic Development. J. Econ. Lit..

[32] Official CMS Webpage. https://www.cms.gov/Research-Statistics-Data-and-Systems/Statistics-Trends-and-Reports/Medicare-Geographic-Variation/GV_PUF.

[33] Center for Medicare and Medicaid Services. https://data.cms.gov/provider-data/dataset/dgck-syfz.

[34] Newhouse J.P., Garber A.M. (2013). Geographic Variation in Medicare Services. N. Engl. J. Med..

[35] Institute of Medicine, Geographic Variation Data Request: A Methodological Overview. https://www.kff.org/wp-content/uploads/sites/2/2011/03/20110217_geographic_variation_methods_paper_-_iom_data.pdf.

[36] Grande G.E., McKerral A., Todd C.J. (2002). Which cancer patients are referred to Hospital at Home for palliative care?. Palliat. Med..

[37] Campbell M., Grande G., Wilson C., Caress A.L., Roberts D. (2010). Exploring differences in referrals to a hospice at home service in two socio-economically distinct areas of Manchester, UK. Palliat. Med..

[38] USAFacts Data. https://usafacts.org/articles/detailed-methodology-covid-19-data/.

[39] CMS Hospice Data. https://www.cms.gov/Medicare/Quality-Initiatives-Patient-Assessment-Instruments/Hospice-Quality-Reporting/Current-Measures.

[40] Census Bureau Quick Facts. https://www.census.gov/quickfacts/fact/table/US/PST045219.

[41] The United States Census Bureau. https://www.census.gov/data/datasets/time-series/demo/popest/2010s-counties-total.html#par_textimage_70769902.

[42] Koenker R., Bassett G. (1978). Regression quantiles. Econom. J. Econom. Soc..

[43] Lamarche C.E. (2006). Quantile Regression for Panel Data. Ph.D. Dissertation.

[44] Buchinsky M. (1995). Quantile regression, Box-Cox transformation model, and the U.S. wage structure, 1963–1987. J. Econom..

[45] Skrondal A., Rabe-Hesketh S. (2004). Generalized Latent Variable Modeling: Multilevel, Longitudinal, and Structural Equation Models.

[46] Raudenbush S.W., Bryk A.S. (2002). Hierarchical Linear Models: Applications and Data Analysis Methods.

[47] Rabe-Hesketh S., Skrondal A. (2012). Multilevel and Longitudinal Modeling Using Stata.

[48] Breiman L. (2001). Random forests. Mach. Learn..

[49] Zou R.Y., Schonlau M. (2019). RFOREST: Stata Module to Implement Random Forest Algorithm.

[50] Frank E., Hall M.A., Witten I.H., Pal C.J. (2016). The WEKA workbench online appendix. Data Mining: Practical Machine Learning Tools and Techniques.

[51] Zhang G.Y., Zhang C.X., Zhang J.S. (2010). Out-of-bag estimation of the optimal hyperparameter in SubBag ensemble method. Commun. Stat. -Simul. Comput..

[52] Experton B., Tetteh H.A., Lurie N., Walker P., Elena A., Hein C.S., Schwendiman B., Vincent J.L., Burrow C.R. (2021). A Predictive Model for Severe COVID-19 in the Medi-care Population: A Tool for Prioritizing Primary and Booster COVID-19 Vaccination. Biology.

[53] Lipovetsky S., Conklin M. (2001). Shapley regression values: Analysis of regression in game theory approach. Appl. Stoch. Models Bus. Ind..

